# Genome-Wide Data-Mining of Candidate Human Splice Translational Efficiency Polymorphisms (STEPs) and an Online Database

**DOI:** 10.1371/journal.pone.0013340

**Published:** 2010-10-11

**Authors:** Christopher A. Raistrick, Ian N. M. Day, Tom R. Gaunt

**Affiliations:** 1 Bristol Genetic Epidemiology Laboratories, School of Social and Community Medicine, University of Bristol, Bristol, United Kingdom; 2 MRC Centre for Causal Analyses in Translational Epidemiology (CAiTE), School of Social and Community Medicine, University of Bristol, Bristol, United Kingdom; Centre de Regulació Genòmica, Spain

## Abstract

**Background:**

Variation in pre-mRNA splicing is common and in some cases caused by genetic variants in intronic splicing motifs. Recent studies into the insulin gene (*INS*) discovered a polymorphism in a 5′ non-coding intron that influences the likelihood of intron retention in the final mRNA, extending the 5′ untranslated region and maintaining protein quality. Retention was also associated with increased insulin levels, suggesting that such variants - splice translational efficiency polymorphisms (STEPs) - may relate to disease phenotypes through differential protein expression. We set out to explore the prevalence of STEPs in the human genome and validate this new category of protein quantitative trait loci (pQTL) using publicly available data.

**Methodology/Principal Findings:**

Gene transcript and variant data were collected and mined for candidate STEPs in motif regions. Sequences from transcripts containing potential STEPs were analysed for evidence of splice site recognition and an effect in expressed sequence tags (ESTs). 16 publicly released genome-wide association data sets of common diseases were searched for association to candidate polymorphisms with HapMap frequency data. Our study found 3324 candidate STEPs lying in motif sequences of 5′ non-coding introns and further mining revealed 170 with transcript evidence of intron retention. 21 potential STEPs had EST evidence of intron retention or exon extension, as well as population frequency data for comparison.

**Conclusions/Significance:**

Results suggest that the insulin STEP was not a unique example and that many STEPs may occur genome-wide with potentially causal effects in complex disease. An online database of STEPs is freely accessible at http://dbstep.genes.org.uk/.

## Introduction

Alternative splicing of pre-mRNA is common in human and other species [1]. Alternative splicing events have been recognised for their effects on protein coding sequences, because most exons contain protein coding sequence which may therefore be present or absent in the translation product, hence altering the *qualities* of the protein produced [2]. Alternative protein versions may subserve different functions, a classic example being calcitonin expressed from thyroid C cells versus CGRP as a neurotransmitter produced by some nerve cells [3]. Such pre-mRNA splicing may be tissue-specific and is equivalent between all individuals in a population, but some is individual-specific because the alleles of a polymorphism differentially affect splicing through their presence in or effect on splicing sequence motifs. For example, a branch point SNP, rs17266594 leads to the Δ2 isoform of BANK1 protein which lacks a putative IP3R-binding domain, altering B cell signalling and activity and SLE disease risk [4]. Similarly, SNP rs6897932 in a putative exonic splice enhancer in *IL7R* seems to affect exon 6 splicing, amounts of soluble versus membrane-bound IL7R and risks of multiple sclerosis [5] and type 1 diabetes [6].

In a study of the human insulin gene (*INS*), we recognised *in silico*, subsequently confirmed *in vitro* and *in silico* [7], and later *in vivo* by others [8], [9], that polymorphism in a 5′ non-coding intron (separating non-coding exon 1 from coding exon 2) could influence intron retention in the final mRNA. In addition to transcriptional effects, there were major proinsulin expression effects, possibly due to the different 5′ untranslated sequence in the message [10]. Messenger RNA 5′ untranslated sequence upstream of the initiator methionine is well known to be able to influence translational efficiency whether through ease of ribosomal roll through from the 5′ cap [11], through internal ribosomal entry sites [12], or through specific regulatory elements [13]. Since about 30% of (vertebrate) genes have at least one 5′ non-coding exon [14], polymorphisms like the *INS* “-23HphI” (IVS1-6A/T) site affecting mRNA 5′ untranslated sequence but not affecting the protein sequence, represent a potentially substantial contribution to protein *quantitative* trait loci (pQTLs), alongside promoter variants and other possible chromatin based locus control polymorphisms. This hitherto unrecognised class of potentially functional variants we designated as ‘STEPs’ (Splice Translational Efficiency Polymorphisms) [15], a class of polymorphisms in non-coding RNA that may affect splicing such as to alter translational efficiency.

We conducted a genome-wide sequence analysis of 5′ untranslated region (UTR) intron and variant information gathered from online databases to identify possible STEPs. Using computational methods we collected data from publicly available sources to find candidate STEPs in consensus regions of introns and in transcripts displaying alternative splicing around the candidate STEP. We further examined candidate STEPs *in silico* for splice site recognition using sequences containing each SNP allele and for expressed sequence tag (EST) evidence of intron retention where possible. Presented here are results of this search, along with 21 potential intron retaining or exon extending STEPs where allele population frequency and EST data has shown a disproportionate retention of one STEP allele, as well as a spliced form. We also developed an online database, dbSTEP (http://dbstep.genes.org.uk/), with current candidate STEPs annotated.

## Results

Our genome-wide analysis and mining of transcript and variant data found 9944 genes containing 5′ UTR introns and resulted in a database of 3324 candidate STEPs (see dbSTEP summary in Table 1) covering 1820 Ensembl and 1117 Vega genes. SNPs represented a 78% majority of these variants, but a large number of variants were short insertions or deletions (INDELs) and do not have HapMap frequency data available. The polypyrimidine tract (PPT) was the most common intronic region for a candidate STEP, with 1829 variants (1381 SNPs, 424 INDELs and 24 other – e.g. Multi-Nucleotide Polymorphism or mixed). The fewest number of variants appears in the shortest intronic motif, the 3′ acceptor splice site (3′ss), with 326 variants (248 SNPs, 78 INDELs). We found a disproportionate number of candidate STEPs for the 12 nucleotide length of PPT searched using the PWM of Zhang *et al.* [16] (where PPT is range −4 to −15), with 3.03 and 5.61 times more candidates in the PPT than in the 5′ss (half the length of the PPT) and 3′ss (quarter the length of the PPT) regions. For each intron we found a mean allelic score difference (non-consensus probability score difference between each allele of a variant at a position in the intron) of 0.367, with a standard deviation of 0.465 and a much higher maximum difference for a 5′ss candidate STEP. We also calculated a disease impact score for each candidate STEP. The mean disease impact score was 1.473 across all possible candidate STEP-motif pairings, with a standard deviation of 1.171 and a much higher maximum value for a SNP in the PPT.

**Table 1:** Summary of dbSTEP content.

		5′ splice site	3′ splice site	Branch point	Polypyrimidine tract	Total
**Number of candidate STEPs**		603	326	566	1,829	3,324^a^
	**CEU** b	77	25	73	236	411
	**CHB** b	74	20	69	222	385
	**YRI** b	85	23	77	245	430
	**JPT** b	72	22	67	220	381
**SNPs**		511	248	458	1,381	2,598
	**Transitions**	309	174	281	895	1,659
	**Transversions**	199	73	177	483	932
	**Tri-allelic**	3	1	0	3	7
**IN-DELs**		90	78	108	424	700
**Other**		2	0	0	24	26
**Disease impact** c						
	**Mean**	1.638	1.250	1.322	1.490	1.473
	**Std. Dev.**	1.752	1.404	0.676	1.009	1.171
	**Max**	8.838	8.022	3.651	14.918	14.918
	**Min**	0	0.123	0.071	0.036	0
**Allelic Score Difference** c						
	**Mean**	0.633	0.848	0.384	0.194	0.367
	**Std. Dev.**	0.678	0.510	0.508	0.167	0.465
	**Max**	4.070	2.700	2.048	0.780	4.070
	**Min**	0	0	0	0	0

a 7 candidate STEPs appear in different motif regions depending on their location in different transcripts. 36 candidate STEPs appear in different chromosomes depending on dbSNP mapping.

b HapMap data.

c Parameters explained in text.

A summary of dbSTEP content constructed from mining of 59251 5′ UTR intron sequences (22553 Ensembl; 36698 Vega) including that from 33372 transcripts (15056 Ensembl; 18316 Vega) from 9944 genes (9111 Ensembl; 5964 Vega).

Using the above content we scanned all 3324 candidate STEPs for any transcript evidence of 3 different splicing patterns: intron retention, alternative splice site (including extending, shortening or skipping exons) and alternative upstream transcript start. The largest of these groups was the upstream transcript start group, with 2558 Ensembl and dbSNP polymorphisms, followed by the alternative splice site group with 1806. The group with the fewest candidates was the intron retention group, containing 170 variants. Of the 170 variants in retained introns, 24 had HapMap minor allele frequencies (MAFs) available for comparison with allele frequencies found in searches of EST data sets (Table 2).

**Table 2:** Summary of candidate STEPs in alternatively spliced transcripts.

		Intron retention	Alternative splice site	Upstream transcript start
**Candidates**		170	1806	2558
	**with frequencies**	24	231	330
	**in 5′ splice site**	45	362	483
	**in 3′ splice site**	16	188	235
	**in branch point**	12	306	454
	**in polypyrimidine tract**	98	957	1391

A STEP causes intron retention if a transcript exists with an exon completely spanning the intron containing the STEP, activation of an alternative splice site if there exists an intron that incompletely overlaps the intron containing the STEP or activation of an upstream transcript start site if there are no overlapping sequences and other transcripts.

### EST analysis of potential intron retaining or exon extending STEPs

Candidate STEPs relating to intron retaining transcripts were mined using an online BLAST search of dbEST [17] for evidence of intron retention in mRNA. Results from EST mining of 143 SNP-intron matches (a subset of the 170 variants that included INDELs) yielded 92 possible STEPs with EST sequences spanning the whole retained intron, as well as EST sequences matching a 40 base sequence spanning the spliced exon-exon junction (20 bases either side). INDELs were excluded from this analysis due to a lack of population frequency data. Of the 92 SNPs exhibiting spliced and unspliced forms, 11 had HapMap-CEU MAFs (see Table 3). Interestingly, all these 11 cases were polymorphic in either the 5′ss or at least −7 bases from the 3′ss (in the PPT) and no likely cases for intron retention were found in either the branch point or the 3′ss.

**Table 3:** List showing 21 candidate STEPs with evidence of alternative splicing in Ensembl/Vega transcripts, HapMap minor allele frequencies greater than zero and BLAST EST evidence of intron retention (above dotted line) or exon extension (below dotted line).

SNP	Gene	Disease Impact^a^	Region^b^	HapMap MAF (samples)	BLAST EST MAF (samples)	BLAST EST Un-spliced/Spliced
rs4674259	*CXCR2*	1.02	ppt	0.483(120)	0.070(100)	100/4
rs11165878	*BRDT*	1.95	5′ss	0.242(120)	0.800 (20)	20/19
rs2236709	*TTC12*	1.18	5′ss	0.225(120)	1.000 (4)	4/6
rs3087370	*POLD2*	2.26	ppt	0.284(118)	0.125 (96)	96/2
rs2738364	*PPP2R3B*	-	ppt	0.267(120)	1.000 (2)	2/15
rs226380	*A2M*	1.94	ppt	0.450(120)	0.750 (16)	16/3
rs9508887	*PSPC1*	1.41	ppt	0.217(120)	0.333 (99)	99/10
rs915942	*RPL10*	0.76	5′ss	0.057(88)	0.130(100)	100/50
rs4881111	*PITRM1*	1.61	ppt	0.442(120)	1.000 (2)	2/50
rs1859143	*COL25A1*	1.10	5′ss	0.483(120)	0.400 (10)	10/1
rs17173334	*GALNT11*	1.21	5′ss	0.025(120)	0.000 (5)	5/19
rs10912675	*FMO1*	1.09	ppt	0.325(120)	0.600 (20)	20/36
rs2276611	*PPIG*	2.49	5′ss	0.093(118)	1.000 (4)	4/50
rs924900	*ZNF713*	0.68	ppt	0.033(120)	1.000 (3)	3/12
rs2076689	*PLCB1*	1.92	ppt	0.110(118)	0.000 (2)	2/32
rs1045761	*TCEAL7*	-	3′ss	0.467(90)	0.325 (80)	80/29
rs1158867	*PROC*	1.44	ppt	0.375(120)	0.200 (10)	10/4
rs13011342	*PLCD4*	1.54	bp	0.017(120)	0.000 (2)	2/1
rs2277382	*ACVRL1*	1.46	bp	0.075(120)	0.750 (8)	8/9
rs17602686	*GLDN*	-	bp	0.149(114)	0.000 (12)	12/15
rs366577	*ENO3*	1.08	5′ss	0.392(120)	0.091 (99)	99/50

a Parameters explained in text.

b Intronic motif region where the SNP lies: 5′ splice site (5′ss), 3′ splice site (3′ss), branch point (bp) or polypyrimidine tract (ppt).

In addition to the intron retention search, this method was repeated for partial intron retention where an exon was extended to include the STEP. Of 508 candidate STEPs scanned (including the 170 candidate intron retaining STEPs), where the STEP was both intronic and exonic by transcript data, an additional 72 SNPs were found to have evidence of alternative splicing by EST evidence. Only 10 of these carried both HapMap allele frequency data as well as EST evidence of spliced and unspliced forms (see Table 3). This set included 1 3′ss candidate, 2 5′ss candidates, 4 polypyrimidine tract candidates and 3 branch point candidates. Alternative splicing of shorter introns (under 700 bases) was related to a candidate STEP in either the 3-base 3′ss or the 6-base 5′ss, while all branch point candidates related to introns of lengths of 3759, 4774 and 35401.

### Splice site prediction of candidate STEPs

All 2140 splice site and polypyrimidine tract candidate STEP SNPs (4633 SNP-transcript cases) from within 5′ and 3′ end PWMs, run through three splice site prediction tools [18]–[20] resulted in 1980 candidate STEPs (4317 SNP-transcript cases) where an allelic difference between predictions was greater than zero for any prediction tool. A total of 1423 of these candidates resulted in a difference greater than zero in 2 or more splice prediction tools. The most predictions came from Human Splice Finder (3788 SNP-transcripts, average difference 16.19%), followed by a neural network splice site prediction tool, NNSplice (3142 SNP-transcripts, average difference 31.03%) and the least from NetUTR (1468 SNP-transcripts, average difference 37.26%), based on the NetGene2 splice prediction tool and trained on a data set of UTR splice patterns. The distribution of these results for different attributes is shown in Figure S1. NNSplice results in a wide range of difference scores between alleles, unlike HSF, where differences were either quite low (<10%) or quite high (>65%). The majority of cases resulting in a high difference were due to no prediction of a splice site for the alternative allele. There was no correlation between score difference and either GC-richness, intron length or the number of known transcripts for a gene.

### Final results set

Combining these results we found 15 SNPs where there existed evidence of alternative splicing among gene transcripts and in EST databases, as well as differential predictions of splice site quality and HapMap minor allele frequencies. These results represent the most likely candidates among our 3324 candidates in dbSTEP.

## Discussion

The paradigm for this pQTL mechanism has been the human insulin gene and we set out to find other polymorphisms genome-wide that exhibit this effect. Our annotation of human 5′ UTR intron sequences has found many candidate polymorphisms which may cause major effects on the expression level of specific proteins through differential splicing of 5′ non-coding regions in the final mRNA.

Our results show STEPs to be potentially common throughout the genome, with 1820 Ensembl genes occurring in our candidate list and 1117 from the Vega database. It is unsurprising to find more candidate STEPs per length of DNA in the PPT than in other intron motifs as the PPT is both a much longer motif and appears to have a lower specificity by the PWMs. Shorter and higher specificity regions (5′ss and 3′ss) have less candidates per base, with 5′ss candidates (a 6 base consensus) double that of the 3′ss (a 3 base consensus). The number of candidate STEPs near exons causing an upstream transcript start (UTS) is an interesting result, perhaps illustrating that the common scenario of alternative splicing in 5′ untranslated sequences results in an alternative first exon and not so commonly the activation of a cryptic splice site [21] or exon skipping. It may also be an artefact of the high number of fragmented or non-coding Ensembl and Vega transcripts currently available.

We predict some STEPs have an effect on disease, with the top 10 disease impact scores (see methods) ranging from 2.72 to 3.75 exclusive of genes in the HLA region of chromosome 6 [22]. This score is used as a rough estimate to indicate possible disease association through LD with reported SNPs in a collection of genome-wide association studies (see Table S5) where possible. Since the majority of candidate STEPs are not represented or tagged in any of the 16 GWAS data sets in Table S5 and have no background frequency data available, no further conclusions can be made as to their disease impact or their abundance *in vivo*.

### EST analysis

We used expressed sequence tag data analysis, as was done for the *INS* gene [7], to provide an initial validation *in silico* of whether alternative splicing around a candidate STEP is seen often *in vivo*.

One prediction was that polymorphisms at “obligate” splice site consensus bases could have a stronger affect on intron retention by causing splicing and non-splicing alleles and a more definitive change in splicing efficiency. Of the final set of intron retaining or exon extending gene transcript suggested by EST and prediction evidence (Table 3), 3 of 21 STEPs are from obligate 5′ss bases (*TTC12*, *GALNT11* and *PPIG*). In both *TTC12* and *GALNT11* cases there is evidence that the efficient splicing allele is retained, suggesting other effects. In the case of *PPIG* we find only retention of the minor allele, in this case the non-consensus A. Despite only 4 EST samples displaying exon extension, this candidate lies at an obligatory splice site position (G allele necessary for splicing) with a rare MAF and 50 EST samples showing the spliced form, suggesting an effect on splicing.

Another prediction was that intron retention would have more biological impact as retention is likely to involve many more nucleotides than other alternative splicing patterns, extending the 5′ UTR by a greater length and therefore exhibiting a more substantial effect on translation. Table 3 shows a disease impact score (see methods) as an estimate of disease association for a given SNP. Many SNPs appear to show a modest effect where tagged and many of these SNPs were not sufficiently tagged in all GWAS datasets. Five candidate STEPs scored highly for disease impact: Peptidyl-prolyl isomerise G (*PPIG*), DNA polymerase delta 2 (*POLD2*), bromodomain testis-specific (*BRDT*) and alpha-2-macroglobulin (*A2M*).

Peptidyl-prolyl isomerise G (*PPIG*), also known as Cyclophilin G, is one of 16 known human cyclophilins involved in protein folding. The precise function of Cylcophilin G is unknown [23], but has been experimentally shown to interact with CDC-Like kinase (*CLK*) and Pinin (*PNN*), two serine/arginine-rich (SR) proteins that are essential for pre-mRNA splicing [23]. Pinin interacts with RNA-binding protein S1 (RNPS1), known to regulate alternative splicing [24]. Previous research has suggested that Cyclophilin G could bind to other SR proteins before being transported into the nucleus, located to nuclear speckles and assist in the folding of the spliceosome [25]. Previous research [10] suggests that UTR length may affect Cyclophilin G expression, which in turn might affect the efficiency of the spliceosome and result in differential levels of alternatively spliced genes. Our candidate STEP is located in the 5′ss of a 5′ UTR intron and by transcript and EST evidence may result in the extension of exon 1, varying UTR length and potentially Cyclophilin G expression.

Another high disease score was in DNA polymerase delta 2 (*POLD2*), part of the DNA polymerase delta complex of 4 proteins which are involved in DNA replication and repair [26], [27]. DNA damage is widely thought to be related to cancer, aging and neurological diseases [28]–[30]. This suggests that any candidate STEPs for *POLD2*, which may cause a change in splicing efficiency leading to differential protein expression, might affect the efficiency of DNA repair and over a prolonged period affect the risk of various degenerative diseases. *POLD2* contains a candidate STEP T>C polymorphism in the PPT of intron 2, where the minor allele C would be expected to splice less efficiently by consensus. However, EST evidence shows splicing to occur rarely in comparison to the unspliced form, suggesting that other factors may contribute to intron retention (such as a weak 5′ss which may lead to the activation of a 3′ss in the upstream exon). Other polymorphisms, e.g. rs3217947 - a T/G polymorphism in the 5′ss of intron 1, may also exhibit an effect in *POLD2*, but lack population frequency data.

Bromodomain testis-specific (*BRDT*) is a protein similar to the RING3 protein family and is thought to have a role in transcriptional regulation in spermatogenesis [31]. Our results showed a large increase in the MAF of rs11165878, perhaps due to a lesser binding efficiency of the G allele to the spliceosome, which may cause an increased rate of intron retention in an already weak motif coupled with a relatively weak PPT (45% pyrimidine). There is evidence that polymorphisms in this region can cause a greater effect than polymorphisms at +3, +5 and +6 (beyond the obligatory GT start) or can have a differential effect dependant on coupling patterns of 5′ss bases [32]. No strong association was found for this SNP (or others in high LD with it) in 16 GWAS data sets (Table S5). Since this gene is reported to have a potential role in spermatogenesis its role in testicular cancer would be of interest, but it was not reported as one of the top GWAS hits for testicular cancer in Rapely *et al.* [33].

Finally, alpha-2-macroglobulin (*A2M*) contains a T>G polymorphism in the PPT which may lead to intron retention in the final mRNA. Other studies into A2M have suggested a relation to Alzheimer's Disease [34]–[37]. Our candidate STEP, rs226380 was not found to have any strong relationship to Alzheimer's Disease in any population studies where it was genotyped [36], although other SNPs in linkage disequilibrium with this potential STEP have (e.g. rs669, D' = 1, r2 = 0.634) [37].

Unfortunately as the EST data are not traceable to specific populations or to specific individuals, these data are purely indicative of intron sequence retention and serve as a reference for alleles known to be retained. The search for STEPs in this way is also limited by candidate STEPs having to be retained in the mRNA product. Many of the 469 candidate STEPs not reported to have EST evidence of alternative splicing may exhibit an effect leading to an upstream transcript start (where an upstream exon is added by activation of an upstream splice site), intron extension (by activation of a weaker splice site in the adjacent exon) or exon skipping.

### Splice site prediction

The use of splice site predictions serves as extra validation of potential STEPs. Although 1980 candidate STEPs were reported to have a score difference greater than zero, we retained for further analysis only the set that had 2 or more score differences greater than zero from the three prediction tools to eliminate the effects of over-prediction from any one prediction tool. Figure S1 shows graphs of score difference values against other attributes potentially descriptive of intron splicing. Zhang *et al.* [16] suggested that GC-richness had an effect on consensus splice site sequences, deriving two PWMs, one for GC-rich introns and the other for GC-poor. Our graph shows no correlation between GC-richness and score difference for any splice prediction tool, suggesting that for a given candidate STEP, there is no differential chance of an effect for GC-richness. Equally, one might expect that the number of transcripts relating to a gene might relate to score difference for a candidate STEP where STEPs are common, perhaps through weaker splice sites facilitating alternative splicing. Again no correlation was found. Finally, we checked for a relation between score difference and intron length, where longer introns may have weaker splice sites as longer sequences increase the probability of having additional splice sites, but no correlation was found.

**Figure 1:**
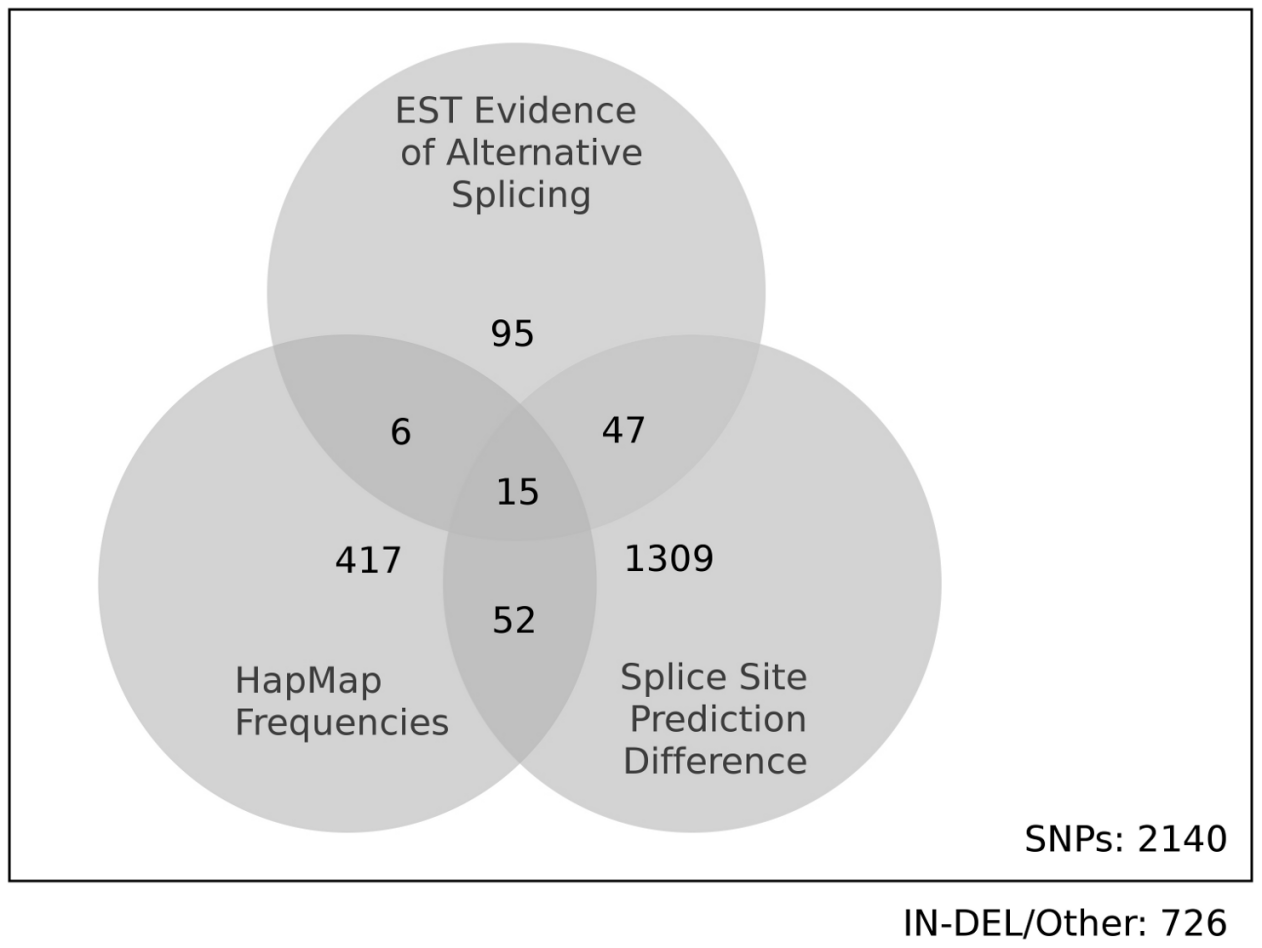
A Venn diagram of candidate STEP SNPs from our search. The circles contain all the candidate STEPs that have HapMap frequencies, all of the candidate STEPs with evidence of either intron retention of exon extension or scored greater than zero in two or more splice site prediction tools.

A large proportion of candidate STEPs with EST evidence of differential splicing also had score differences for 2 or more splice prediction tools. A final set of 15 candidates was returned with positive results for both search methods and population frequency data to check for a real SNP. As we can see from Figure 1, there was a lack of unity between the set of HapMap SNPs and the SNPs available with EST evidence. Potentially many more of the remaining 142 SNPs with EST evidence, but no HapMap frequencies could be shown as putative STEPs if these data became available. Better *in silico* prediction may be obtained with greater understanding of the splicing process and in particular how a STEP might inhibit the splicing machinery. Table 4 displays the final 15 STEPs (a subset of Table 3) requiring further follow-up.

**Table 4:** Table of candidate STEPs with EST evidence (Table 3) and splice site prediction.

SNP	Gene	Average Difference
rs2276611	*PPIG*	**74.28**
rs2236709	*TTC12*	**45.34**
rs1045761	*TCEAL7*	**22.83**
rs11165878	*BRDT*	**22.49**
rs9508887	*PSPC1*	**19.73**
rs2738364	*PPP2R3B*	**17.51**
rs226380	*A2M*	**10.55**
rs915942	*RPL10*	**07.25**
rs4881111	*PITRM1*	**06.90**
rs13011338	*ZNF142*	**03.45**
rs17207454	*MSH5*	**02.14**
rs924900	*ZNF713*	**01.54**
rs10912675	*FMO1*	**01.30**
rs4674259	*CXCR2*	**00.37**
rs2076689	*PLCB1*	**00.35**

A change in splice site recognition score was recorded in 2 or more prediction tools for each candidate. For cases where no splice site was detected this is asserted as 0 for the purposes of deriving a difference score.

### dbSTEP

The database of candidate STEPs (dbSTEP, http://dbstep.genes.org.uk/) provides a resource for further follow-up of the novel genetic mechanism of splice translational efficiency polymorphisms. Our database facilitates searches by gene, rs number, gene and transcript IDs (Ensembl and Vega) or chromosomal region, as well as filtering by motif region and variant type (SNP or INDEL). Searches return a list of all possible transcript-intron-STEP matches, which link to summary pages of annotations for each candidate STEP and links to relevant external resources. Successive variant frequency and transcript data updates will add to this resource and aim to validate more candidate STEPs.

### Future work

This study focused on human 5′ non-coding intron 5′ splice sites, 3′ splice sites (extending into the polypyrimidine tract) and branch point sites. There is the potential to extend these predictions to splice enhancers and silencers which would affect the mRNA 5′ leader; and to extend the database to other living species using the same process which generated dbSTEP. Improvements in SNP databases, 5′ exon identifications and motif predictions will all enable updates. Cross-reference annotation using a 5′ UTR functional elements database [13] may be useful as knowledge of translational control expands.

## Materials and Methods

### Data collection

**Figure 2:**
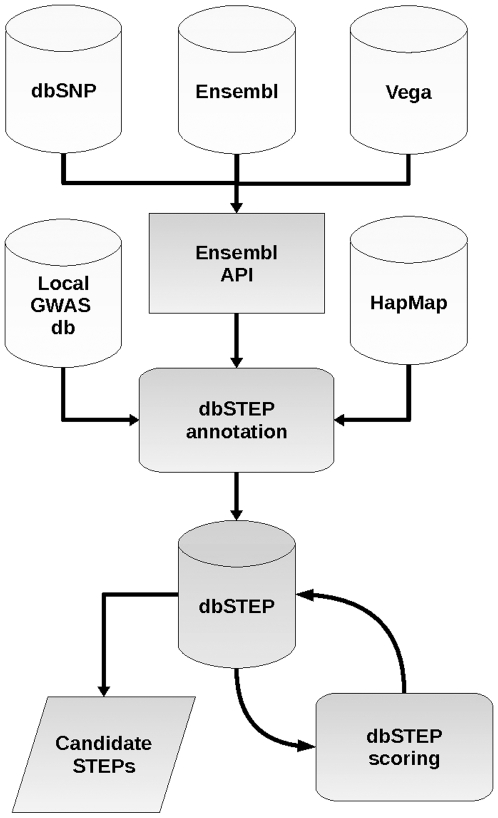
Diagram showing data sources and flow in generating dbSTEP. Variant and transcript information is collected through the Ensembl API (Application Programming Interface) and annotated along with other data collected from HapMap and local GWAS (Genome-Wide Association Study) repositories. Data in dbSTEP are then scored and mined for candidate STEPs (Splice Translational Efficiency Polymorphisms).

Data were collected using a suite of Perl scripts (available from the authors on request), accessing Ensembl and Vega via the Perl API (Bio::EnsEMBL) and other sources (dbSNP and HapMap) via an automated user script to access each relevant website, to construct a database using MySQL (software version MySQL 5.0.67-0ubuntu6). This database was named dbSTEP (Figure 2). Ensembl [38] provided all necessary variant (SNP and small insertion/deletion) and transcript data for this project and was synchronised regularly to remain up-to-date. Where available, gene transcripts were also downloaded from the Vertebrate Genome Annotation (Vega) database [39] at the Wellcome Trust Sanger Institute, which provides a database of manually curated transcript information aligned to a different chromosome build from Ensembl (as of build 36). Data were entered into a core relational database by chromosome, comprising tables of Ensembl and Vega transcripts, tables of corresponding transcript-exons and transcript-introns and tables of genetic variants. Tables were then queried to create a public database with a subset of each table in the core database, containing only candidate STEPs (i.e. variants in conserved splicing sequences of a 5′ UTR intron of a transcript), their related transcript-introns and related flanking transcript-exons. Finally, allele frequency data downloaded from HapMap [40] were mapped to candidate variants in dbSTEP (HapMap phase 3).

### Annotation

Transcript sequences in dbSTEP were annotated according to published consensus patterns and motifs. Table S1 lists annotations made to transcript exons and introns for use in the online database. The first of these is a count of exonic splice enhancers (ESEs) [41], which gives the total occurrences of all candidate ESEs in a given exon, taken from a previously published list of hexamers. Other annotations include counts for each intron of: G-triplets [42], [43], a previously suggested intronic splicing enhancer (ISE), and G-triplet pairs (pairs of GGG sequences fewer than 4 nucleotides apart).

The 5′ donor splice site, 3′ acceptor splice site and branch point are key motifs in describing an intron and determining the binding efficiency to the spliceosome. Previous research has identified consensus sequences at both ends of each intron [21], [44]. Almost all introns start with the dinucleotide sequence GT and end AG by the “obligate” GT-AG rule [45] (with the exception of very rare GC-AG and AT-AC splicing [46], [47]) and have specific base preferences at a number of adjacent bases. Most polymorphisms in a GT-AG sequence should result in no splicing at that site for one allele. Here we have extracted 6 bases at the intron 5′ end to represent the donor splice site and 15 bases at the intron 3′ end to represent the acceptor splice site and part of the polypyrimidine tract (PPT) for each intron. Recent research has derived a consensus sequence of YTNAY (where N denotes any base A, C, G or T) for the branch point in human, a short sequence towards the end of an intron which recruits elements of the spliceosome to begin the two step splicing process [48]. Introns were scanned for branch point consensus matches within a region 21 to 34 bases upstream of the 3′ intron end according to Gao *et al.* [48]. Lastly, introns were analysed for percentage GC and PPT richness. GC-rich introns were scored separately from GC-poor introns in scoring matrices in line with varying isochores [16], [49]. PPT richness influences recruitment of the polypyrimidine tract binding protein (PTB), affecting alternative splicing [50], [51].

### Scoring

For evaluating possible absolute or relative efficiencies of intron removal from the final mRNA, allelic score differences were calculated for 5′ splice, 3′ splice and branch point consensuses (respectively 6, 15 and 5 base lengths) by computing the difference in base likelihood for the allelic bases observed at the relevant base position in the consensus. Thus a change involving an obligate base would score 1, a transition at a random position irrelevant to the consensus would score 0.1, since average AT base pair frequency is 0.4, average CG base pair frequency 0.6 in the human genome. The position weight matrices (PWMs) of Zhang *et al.* [16] (which also take account of intron percentage GC-richness above or below 50%) were used for this calculation for intron boundaries and the PWM of Gao *et al.* [48] was used for branch points (see Tables S2, S3, S4). It was assumed that the importance of location in the effect on the splice consensus is already represented in the relative bias toward usage of a specific base at that position, i.e. that periodicity and coupling effects for nearby bases are minor [52].

For evaluating possible STEP effects across a range of common diseases SNP data were cross referenced to publicly released genome-wide association data (Table S5) using linkage disequilibrium information in HapMap [40] to identify nearby SNPs in allelic association with the candidate STEP. Graphical plots show -log_10_(p-value) versus linkage disequilibrium r^2^ for each relevant SNP within publicly available data (for thirteen separate diseases, see Table S5). This was implemented in a local MySQL database and will be expanded as and when such studies can make p-value data available. To provide an immediate indicator of disease/functional relevance, the highest product of r^2^ and -log_10_(p-value) (“disease impact”) is calculated as a descriptive disease score for the 13 diseases and is displayed in dbSTEP search results. This score provides an estimate of disease association by using the linkage disequilibrium score (range 0 to 1, where 1 is perfect linkage disequilibrium) as a weighting for the p-value for a given SNP.

### Splicing prediction

**Figure 3:**
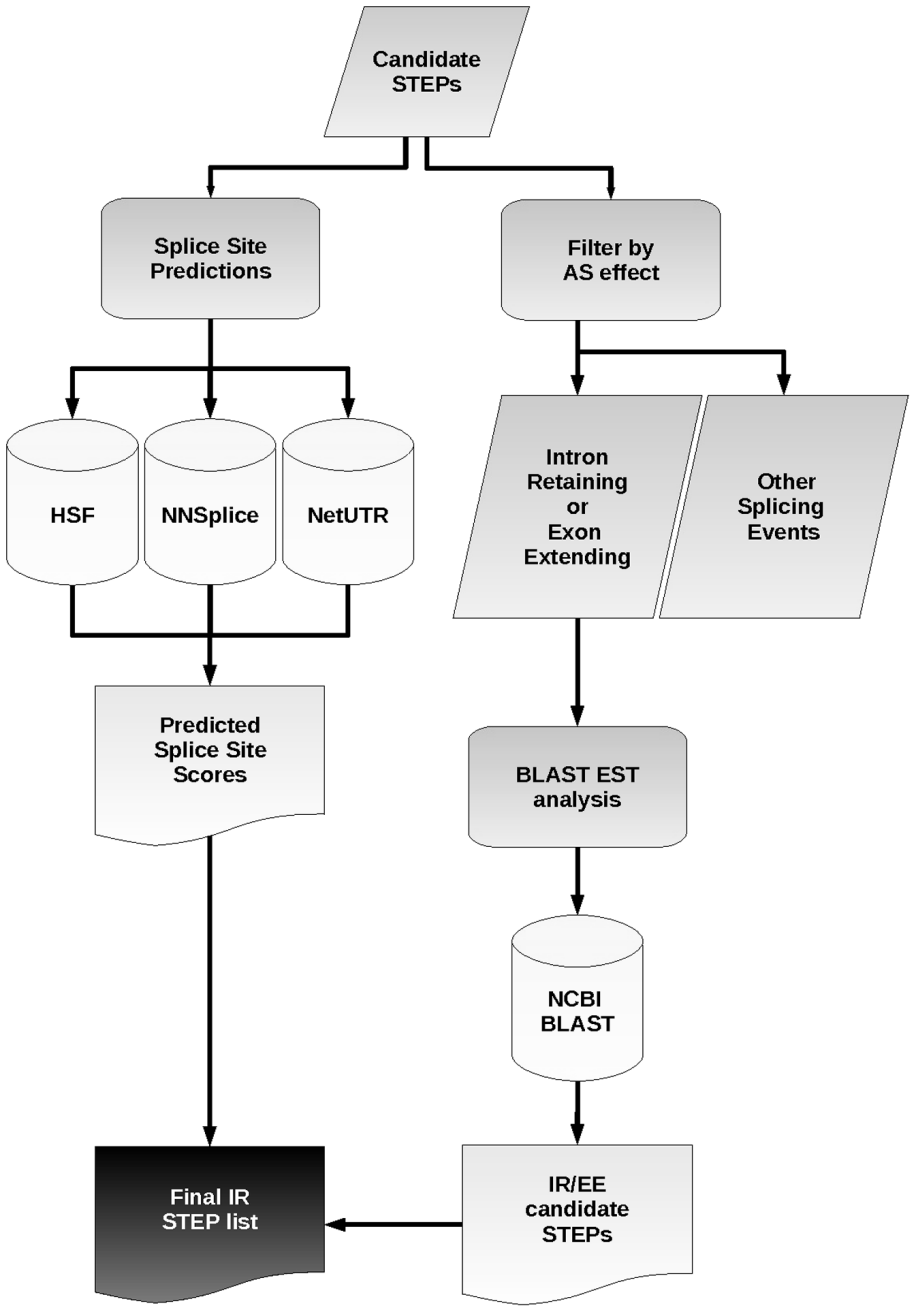
Diagram showing data flow in deriving a final potential STEP list. Candidate STEPs were scanned for splice site prediction differences via 3 online tools as well as filtered for candidates which are retained (causing full intron retention of exon extension) and checked via a BLAST search of EST databases. These results are then combined to derive a final intron retaining STEP list.

Candidate STEPs alleles were systematically submitted in the sequence context of their transcript-intron to a series of online splicing prediction algorithms to generate an *in silico* prediction of splicing efficiency via recognition of the 3′ and 5′ splice sites (Figure 3). Sequences for each SNP allele covering 60 nucleotides around a transcript splice site (20 exonic, 40 intronic) were scanned using three splice site prediction tools: Human Splicing Finder (version 2.4) [18], NNSPLICE (version 0.9) [19] and NetUTR (version 1.0b) [20]. Splice site sequences, instead of full intron sequences, were scanned as the length of some introns exceeded the limit allowed by all splice site prediction tools. A Perl script was used to build each 60 nucleotide sequence from the database and submit to the relevant online tools before analysing the results. Results returned for each submitted sequence were checked for the correct prediction of the splice site and prediction scores submitted to dbSTEP. Where the transcript splice site was not predicted no score was recorded. A difference score between SNP alleles was derived, the difference being equal to the predicted splice site score where a splice site was not found for the opposing allele.

### EST mining

**Figure 4:**
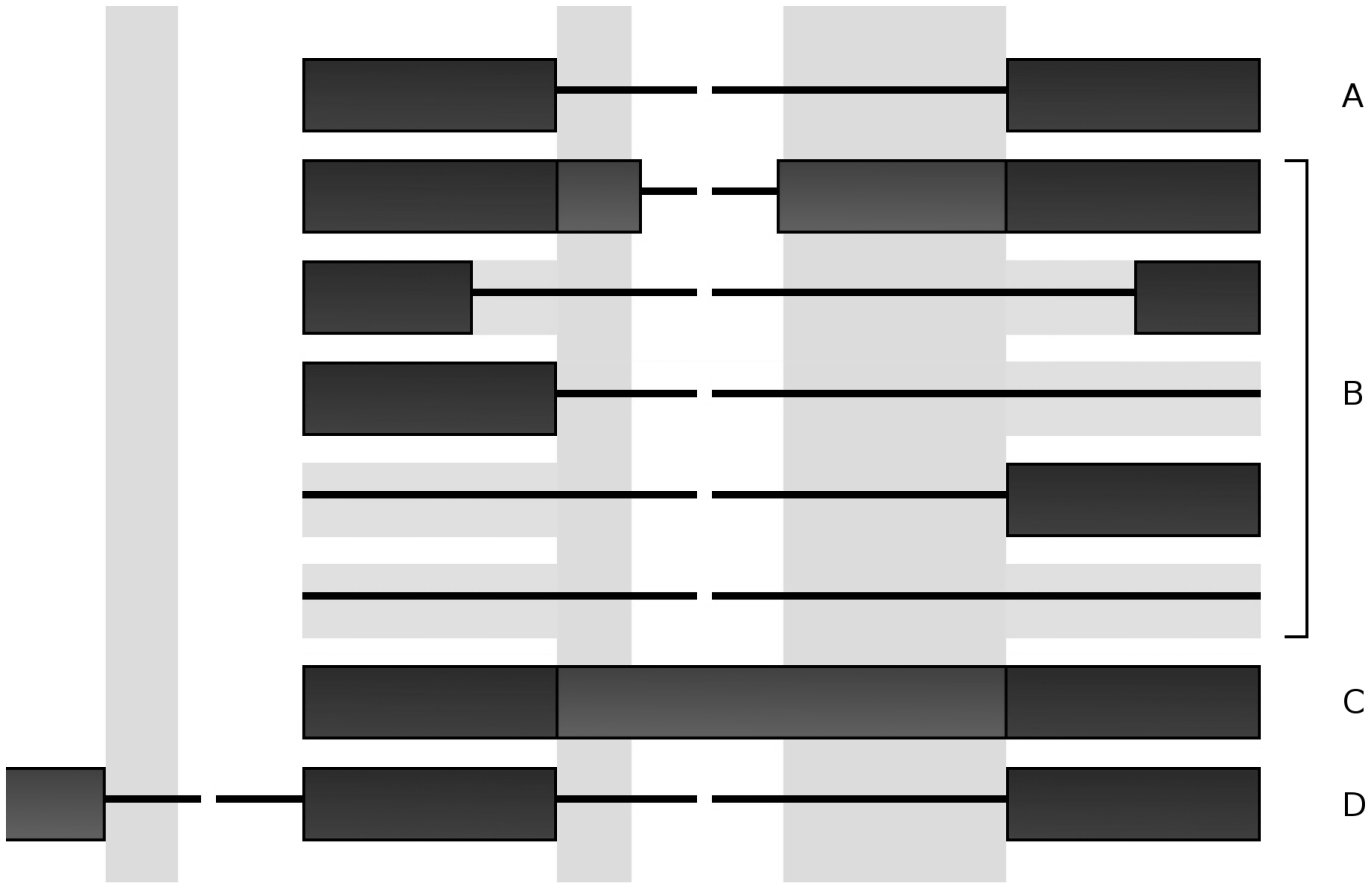
Box diagram of aligned alternative splicing patterns in relation to a given splice form. **A.** Boxes are exonic and lines are intronic. Vertical bands denote splice site motif regions where a STEP may occur. A shows a standard splice form with a single intron. B shows partial intron retention or extension of either exon, as well as exon skipping. C shows full intron retention. D shows an upstream exon activated by a splice site.

Using the list of candidate STEPs obtained through SNP and gene transcript data, exon structure around each candidate was scanned to split the list into those that are possibly intron retaining (exon spans the whole intron), splice to alternative splice sites (introns exist that overlap intron with SNP, but do not share the same start and end positions) or cause an upstream transcript start (no overlapping introns or exons) (see Figure 4). In addition to candidate STEPs in the intron retaining group, all STEPs that were found to be both intronic and exonic by transcript evidence were checked for EST evidence of splicing and partial intron retention.

We used the list of intron retaining candidate STEPs and each corresponding intron sequence that may be retained to search EST databases for evidence of intron retention *in vivo* (Figure 3). A search for RNA-Seq libraries for evidence of spliced and unspliced forms yielded none in the public domain. Using Perl scripts we queried the online NCBI BLAST website (http://blast.ncbi.nlm.nih.gov/) [53] and submitted fragments of sequence for alignment against dbEST [17]. For each STEP-intron case regarding complete intron retention, we submitted 2 whole intron sequences (one for each allele of the STEP) and another 40-base sequence spanning the exon-exon junction of the spliced form (where the junction exists between bases 20 and 21). For partially retained sequences two 41-base sequences (20 flanking candidate STEP) were submitted along with the 40-base exon-exon junction. Intron and exon sequences were taken from one Ensembl or Vega transcript in each case where the STEP was predicted to have an effect. Results received from the online BLAST search were used where the resulting subject sequence was the full length of the query sequence, with gaps allowed. Each intronic subject sequence was parsed for which allele occurred for our STEP and the quantity recorded along with the quantity of spliced forms. If a given STEP-intron case had evidence of the spliced form as well as a bias in the number of STEP alleles relative to population allele frequency in the intron sequence it was shortlisted as a putative STEP (Table 3).

### dbSTEP online database

An online interface to the dbSTEP database was created using Perl common gateway interface (CGI) scripts running under Apache2 (http://www.apache.org/) on a Linux webserver. The interface was designed to provide user-friendly graphical access to the MySQL database generated by our genome-wide STEP analysis, and is accessible at http://dbstep.genes.org.uk/.

## Supporting Information

Table S1 Annotations made on transcript introns.
(0.13 MB PDF)

Table S2 Scoring matrix for 5' donor splice sites based on matrices from Zhang et al. (1998), where 1 is the first base of the intron and the scores are the probability of a base not appearing at a position.
(0.17 MB PDF)

Table S3 Scoring matrix for 3′ acceptor splice sites based on matrices from Zhang et al. (1998), where -1 is the last base of the intron and the scores are the probability of a base not appearing at a position.
(0.18 MB PDF)

Table S4 Scoring matrix for branch points based on matrices from Gao et al. (2008), where 0 is the location of the branch point and the scores are the probability of a base not appearing at a position.
(0.16 MB PDF)

Table S5 List of 16 GWAS data sets used to look for disease association. These data sets were downloaded from the study website upon their original release.
(0.14 MB PDF)

Figure S1 A, B and C show plots of allele difference scores from in silico prediction tools against 3 different attributes which may relate to STEPs: Number of transcripts (A), GC-richness (B) and intron length (C). Blue points are NNSplice results, green points are NetUTR results and red points are HSF results.
(0.36 MB PDF)
