# Supplementary material for: Genome-Wide Data-Mining of Candidate Human Splice Translational Efficiency Polymorphisms (STEPs) and an Online Database
Source: PLoS One. 2010 Oct 11;5(10):e13340. doi: 10.1371/journal.pone.0013340 (PMC2952627; doi:10.1371/journal.pone.0013340)
Supplement: Table S1 — Annotations made on transcript introns. (0.13 MB PDF) [file pone.0013340.s001.pdf]

Table S1: Annotations made on transcript introns.

**Annotation**	**Details**
Candidate ESE Count	Counts the total number of candidate Exonic Splicing Enhancer hexamers in exon sequences.
G triplet count	Counts the number of "GGG" sequences - a putative intronic splicing enhancer (ISE) - in an intron.
G triplet pairs count	Counts the number of "GGG" sequence pairs of 0 to 4 nucleotides apart in an intron.
Polypyrimidine Tract	Records the sequence, length and percentage or pyrimidines between 3' intron end and the Branch Point.
GC %	Records the percentage of nucleotides that are C or G in an intron.
5' Splice Site	Records the 6 base sequence at the 5' end of an intron and whether it matches the consensus GTRAGT.
3' Splice Site	Records the 3 base sequence at the 3' end of an intron and whether it matches the consensus YAG.
Branch Point	Records, where matching consensus YTNAY, a sequence of 5 nucleotides between 21 and 34 bases of the 3' intron end.
