# Supplementary material for: Genome-Wide Data-Mining of Candidate Human Splice Translational Efficiency Polymorphisms (STEPs) and an Online Database
Source: PLoS One. 2010 Oct 11;5(10):e13340. doi: 10.1371/journal.pone.0013340 (PMC2952627; doi:10.1371/journal.pone.0013340)
Supplement: Table S2 — Scoring matrix for 5' donor splice sites based on matrices from Zhang et al. (1998), where 1 is the first base of the intron and the scores are the probability of a base not appearing at a position. (0.17 MB PDF) [file pone.0013340.s002.pdf]

Table S2: Scoring matrix for 5' donor splice sites based on matrices from Zhang *et al.* (1998), where 1 is the first base of the intron and the scores are the probability of a base not appearing at a position.

Low GC Introns (under 50%)	**1**	**2**	**3**	**4**	**5**	**6**
**A**	1.00	1.00	0.29	0.27	0.89	0.79
**C**	1.00	1.00	0.98	0.94	0.94	0.90
**G**	0.00	1.00	0.76	0.92	0.25	0.86
**T**	1.00	0.00	0.97	0.87	0.92	0.45
High GC Introns (over 50%)	**1**	**2**	**3**	**4**	**5**	**6**
**A**	1.00	1.00	0.62	0.30	0.95	0.87
**C**	1.00	1.00	0.96	0.91	0.95	0.79
**G**	0.00	1.00	0.44	0.86	0.14	0.75
**T**	1.00	0.00	0.98	0.93	0.96	0.59
