# Supplementary material for: Genome-Wide Data-Mining of Candidate Human Splice Translational Efficiency Polymorphisms (STEPs) and an Online Database
Source: PLoS One. 2010 Oct 11;5(10):e13340. doi: 10.1371/journal.pone.0013340 (PMC2952627; doi:10.1371/journal.pone.0013340)
Supplement: Table S3 — Scoring matrix for 3′ acceptor splice sites based on matrices from Zhang et al. (1998), where -1 is the last base of the intron and the scores are the probability of a base not appearing at a position. (0.18 MB PDF) [file pone.0013340.s003.pdf]

Table S3: Scoring matrix for 3' acceptor splice sites based on matrices from Zhang *et al*. (1998), where -1 is the last base of the intron and the scores are the probability of a base not appearing at a position.

Low GC Introns (under 50%)	**-15**	**-14**	**-13**	**-12**	**-11**	**-10**	**-9**	**-8**	**-7**	**-6**	**-5**	**-4**	**-3**	**-2**	**-1**
**A**	0.85	0.86	0.87	0.89	0.90	0.90	0.89	0.88	0.87	0.89	0.90	0.74	0.93	0.00	1.00
**C**	0.76	0.79	0.80	0.78	0.79	0.78	0.75	0.72	0.72	0.75	0.78	0.75	0.45	1.00	1.00
**G**	0.90	0.88	0.90	0.91	0.90	0.91	0.90	0.90	0.92	0.95	0.95	0.85	0.99	1.00	0.00
**T**	0.49	0.47	0.43	0.42	0.41	0.41	0.46	0.50	0.49	0.41	0.37	0.67	0.63	1.00	1.00
High GC Introns (over 50%)	**-15**	**-14**	**-13**	**-12**	**-11**	**-10**	**-9**	**-8**	**-7**	**-6**	**-5**	**-4**	**-3**	**-2**	**-1**
**A**	0.90	0.92	0.93	0.92	0.94	0.94	0.96	0.92	0.92	0.93	0.94	0.81	0.98	0.00	1.00
**C**	0.59	0.58	0.59	0.60	0.62	0.57	0.58	0.54	0.51	0.46	0.55	0.62	0.18	1.00	1.00
**G**	0.85	0.86	0.86	0.87	0.87	0.88	0.87	0.86	0.90	0.92	0.92	0.74	1.00	1.00	0.00
**T**	0.66	0.64	0.62	0.61	0.57	0.61	0.59	0.68	0.67	0.69	0.59	0.83	0.84	1.00	1.00
