# Supplementary material for: Genome-Wide Data-Mining of Candidate Human Splice Translational Efficiency Polymorphisms (STEPs) and an Online Database
Source: PLoS One. 2010 Oct 11;5(10):e13340. doi: 10.1371/journal.pone.0013340 (PMC2952627; doi:10.1371/journal.pone.0013340)
Supplement: Table S4 — Scoring matrix for branch points based on matrices from Gao et al. (2008), where 0 is the location of the branch point and the scores are the probability of a base not appearing at a position. (0.16 MB PDF) [file pone.0013340.s004.pdf]

Table S4: Scoring matrix for branch points based on matrices from Gao *et al*. (2008), where 0 is the location of the branch point and the scores are the probability of a base not appearing at a position.

	**-5**	**-4**	**-3**	**-2**	**-1**	**0**	**1**	**2**	**3**
**A**	0.746	0.768	0.917	0.934	0.834	0.077	0.818	0.698	0.799
**C**	0.790	0.773	0.530	0.840	0.552	0.967	0.669	0.726	0.609
**G**	0.746	0.807	0.873	0.972	0.823	0.983	0.934	0.888	0.888
**T**	0.718	0.652	0.680	0.254	0.790	0.972	0.580	0.687	0.704
