# Supplementary material for: Genome-Wide Data-Mining of Candidate Human Splice Translational Efficiency Polymorphisms (STEPs) and an Online Database
Source: PLoS One. 2010 Oct 11;5(10):e13340. doi: 10.1371/journal.pone.0013340 (PMC2952627; doi:10.1371/journal.pone.0013340)
Supplement: Table S5 — List of 16 GWAS data sets used to look for disease association. These data sets were downloaded from the study website upon their original release. (0.14 MB PDF) [file pone.0013340.s005.pdf]

Table S5: List of 16 GWAS data sets used to look for disease association. These data sets were downloaded from the study website upon their original release.

Genome-wide association study	Source	Reference
Bipolar Disorder	Wellcome Trust Case Control Consortium	(The Wellcome Trust Case Control Consortium 2007)
Crohn's Disease	Wellcome Trust Case Control Consortium	(The Wellcome Trust Case Control Consortium 2007)
Coronary Artery Disease	Wellcome Trust Case Control Consortium	(The Wellcome Trust Case Control Consortium 2007)
Rheumatoid Arthritis	Wellcome Trust Case Control Consortium	(The Wellcome Trust Case Control Consortium 2007)
Hypertension	Wellcome Trust Case Control Consortium	(The Wellcome Trust Case Control Consortium 2007)
Type 1 Diabetes	Wellcome Trust Case Control Consortium	(The Wellcome Trust Case Control Consortium 2007)
Type 2 Diabetes	Wellcome Trust Case Control Consortium	(The Wellcome Trust Case Control Consortium 2007)
Breast Cancer	Cancer Genetic Markers of Susceptibility	(Hunter et al. 2007)
Prostate Cancer	Cancer Genetic Markers of Susceptibility	(Yeager et al. 2007)
Colorectal Cancer	Institute of Cancer Research	(Broderick et al. 2007)
Parkinson's Disease	National Institute of Neurological Disorders and Stroke	(Fung et al. 2006)
Age-related Macular Degeneration	Age-Related Eye Disease Study	(Age-Related Eye Disease Study Research Group 1999)
Amyotrophic Lateral Sclerosis	University Medical Center Utrecht	(van Es et al. 2007)
Type 2 Diabetes	Broad Institute	http://www.broadinstitute.org/diabetes
Type 2 Diabetes	FUSION	(Zeggini et al. 2008)
Type 2 Diabetes	Diabetes Genetics Inititative	(Saxena et al. 2007)
