# Supplementary material for: Genome-Wide Data-Mining of Candidate Human Splice Translational Efficiency Polymorphisms (STEPs) and an Online Database
Source: PLoS One. 2010 Oct 11;5(10):e13340. doi: 10.1371/journal.pone.0013340 (PMC2952627; doi:10.1371/journal.pone.0013340)

Figure S1: **A**, **B** and **C** show plots of allele difference scores from *in silico* prediction tools against 3 different attributes which may relate to STEPs: Number of transcripts (**A**), GC-richness (**B**) and intron length (**C**). Blue points are NNSplice results, green points are NetUTR results and red points are HSF results.

A

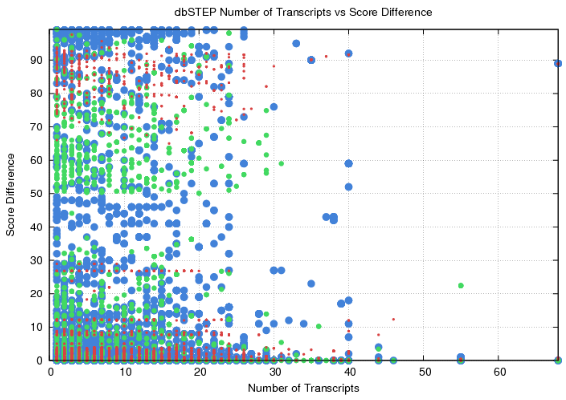

B

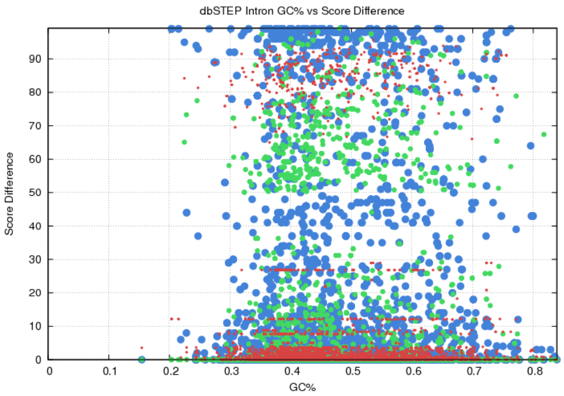

C

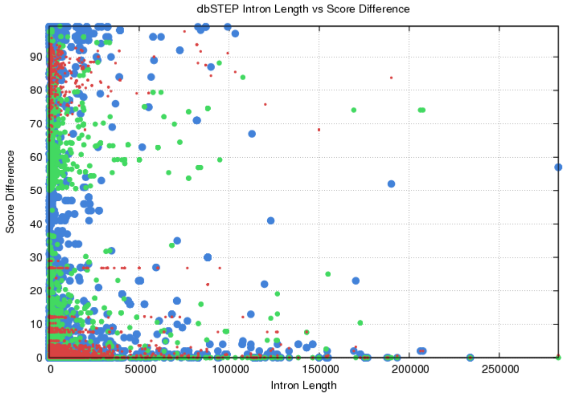

Supplement: Figure S1 — A, B and C show plots of allele difference scores from in silico prediction tools against 3 different attributes which may relate to STEPs: Number of transcripts (A), GC-richness (B) and intron length (C). Blue points are NNSplice results, green points are NetUTR results and red points are HSF results. (0.36 MB PDF) [file pone.0013340.s006.pdf]
